# Spatial and Temporal Variation in Paralytic Shellfish Toxin Production by Benthic *Microseira (Lyngbya) wollei* in a Freshwater New York Lake

**DOI:** 10.3390/toxins11010044

**Published:** 2019-01-15

**Authors:** Zacharias J. Smith, Robbie M. Martin, Bofan Wei, Steven W. Wilhelm, Gregory L. Boyer

**Affiliations:** 1 Department of Chemistry, State University of New York College of Environmental Science and Forestry, Syracuse, NY 13210, USA; bwei101@syr.edu (B.W.); glboyer@esf.edu (G.L.B.); 2 Department of Microbiology, University of Tennessee, Knoxville, TN 37996, USA; rmarti49@vols.utk.edu (R.M.M.); wilhelm@utk.edu (S.W.W.)

**Keywords:** freshwater cyanotoxins, paralytic shellfish poisoning toxins, *Microseira*, *Lyngbya*

## Abstract

Butterfield Lake is a mesotrophic lake in New York State where residents and pets have experienced unexplained health issues. *Microseira wollei* (basionym *Lyngbya wollei*) was found at two of 15 sites in Butterfield Lake and analyzed for microcystins, anatoxins, cylindrospermopsins, and paralytic shellfish poisoning toxins (PSTs). Only PSTs and trace levels of anatoxin-a were detected in these samples. This is the first published report of PSTs within a New York State lake. To evaluate the environmental and temporal drivers leading to the observed toxicity, PST content at the two sites was examined in detail. There were distinct differences in the total PST content, filament nutrient, filament chlorophyll, and relationship to environmental drivers between the sites, as well as distinct differences in the total PST content measured using different analytical techniques. A multivariate model containing site, temperature, and filament chlorophyll explained 85% of the variation in PSTs observed over the growing season. This work emphasizes the importance of proper site selection and choice of analytical technique in the development of monitoring programs to protect lake users from the occurrence of benthic cyanobacteria toxins.

## 1. Introduction

Paralytic shellfish poisoning toxins (PSTs) are a class of algal toxins which inhibit the sodium channel of higher organisms, including humans and marine mammals [1,2,3]. Produced by a variety of algae and cyanobacteria, these toxins create significant human health and economic problems in regions where they occur. Saxitoxin (STX), the parent compound of the PSTs family, is extremely toxic and is closely monitored where there is a high potential for exposure. There are more than 50 known analogs of STX [4]; many are common, but most are less toxic than STX as measured by the mouse bioassay [5,6,7]. Marine PSTs are produced by many dinoflagellate species and lead to illnesses every year through consumption of contaminated fish and shellfish [8,9,10]. PST contamination of shellfish can result in significant economic losses through the closure of shellfish beds and the added costs of monitoring for PSTs [11]. Oral and written records suggest that PST-producing blooms are not new and that poisonings from consumption of contaminated shellfish (known as paralytic shellfish poisoning) have been occurring for centuries [12,13].

PSTs were originally thought to predominately occur in marine environments. This assumption has been challenged as reports of PSTs produced by freshwater cyanobacteria grow in number. Freshwater PST-producing cyanobacteria occur in Australia [14], Brazil [15], United States [5,16,17], Canada [18], Germany [19], Russia [20], and New Zealand [21,22], suggesting a world-wide distribution. PST-producing genera in these countries included *Dolichospermum* (*basionym Anabaena*) (Australia), *Raphidiopsis* (*basionym Cylindrospermopsis*) (Brazil), *Microseira* (*basionym Lyngbya*) (USA and Canada), *Scytonema* (New Zealand), and *Cuspidothrix* (*basionym Aphanizomenon*) (USA). PST production is highly variable within each clade as even closely related species do not always produce toxins [23].

Benthic cyanobacteria produce a number of cyanotoxins including microcystins, anatoxins, cylindrospermopsins, and PSTs [24]. Benthic cyanobacteria and their toxins have been associated with the deaths of dogs in New Zealand [22], France [25], California (USA) [26], and of cows in Switzerland [27]. Benthic PSTs have not been linked with these major exposure events, but PST-producing *Microseira wollei* (basionym *Lyngbya wollei*) [28] have been reported in several freshwater North American water bodies. Within the United States, six new analogs of PSTs referred to as the lyngbyatoxins (LTXs; not to be confused with the dermatoxins with the same name) were purified in 1997 from *M. wollei* collected from the Guntersville Reservoir, Alabama, USA [5]. These new freshwater PSTs were found in the St. Lawrence River near Montreal and in Florida [16,21], but they were not detected in cultures of PST-toxic cyanobacteria from New Zealand and Brazil [29]. Freshwater PST production, as measured by the ELISA assay, is widespread in Ohio, though the extent of the problem and the PST variants involved remains poorly characterized [30]. Benthic cyanobacteria have been observed in a number of New York lakes (personnel observation), but little is known about potential toxin production by benthic cyanobacteria in these water bodies.

**Figure 1:**
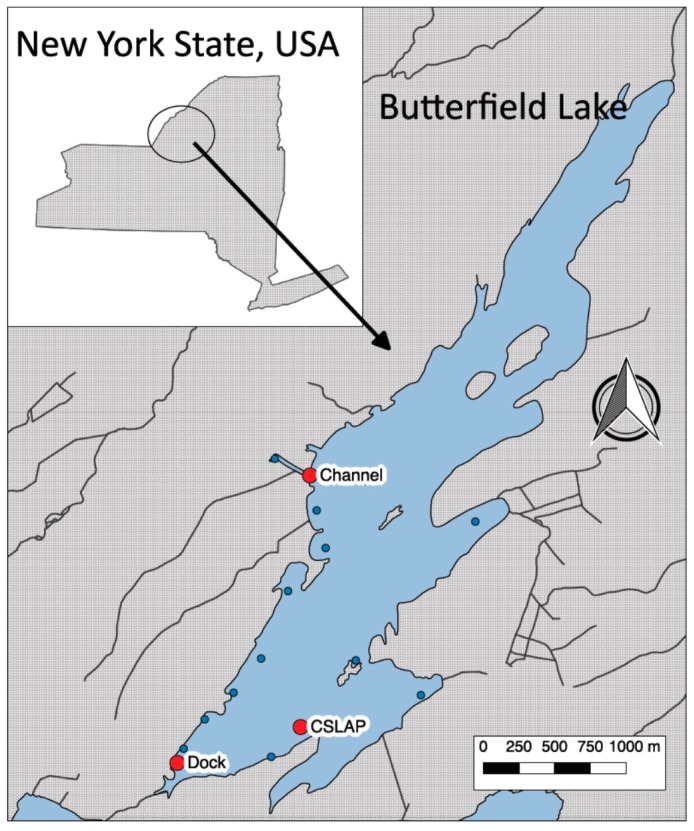
Map of Butterfield Lake (44°19′10.4″ N 75°46′29.0″ W) showing the location of the Channel, Dock and Citizens Statewide Lake Assessment Program (CSLAP) sampling sites. Other sampling sites where *Microseira* or other benthic cyanobacteria were not found are indicated by the blue points. The insert shows the location of Butterfield Lake within New York State.

Butterfield Lake (44°19′10.4″ N 75°46′29.0″ W) is a mesotrophic lake in the foothills of the New York State Adirondack Mountains (Figure 1). The region surrounding the lake is rural and sparsely populated, with small stretches of farmland and a limited number of small towns and scattered individual homes. Butterfield Lake is at the lower end of the Indian River chain of lakes and the watershed area (4419 ha) is mostly forests, shrubs and grasses. For the region, the lake is relatively large (407 ha), with an average depth of 4 m, and is 14 m deep at its deepest point. It has one public access boat launch and two small private marinas on the southwestern shoreline. Butterfield Lake has been part of New York State’s Citizen Statewide Lake Assessment Program (CSLAP) for water quality since 1986 and has been routinely sampled for nutrients, chlorophyll (Chl), and other physical parameters [31]. In the years 2015–2017, the average surface water total phosphorus was 0.014 ± 0.003 mg P/L, with a hypolimnetic total phosphorus average of 0.27 ± 0.08 mg P/L. Surface water total nitrogen and total dissolved nitrogen averaged 0.44 ± 0.02 mg N/L and 0.008 ± 0.001 mg N/L, respectively, over the same time period. Pelagic algae in the lake were likely to be phosphorus limited as the pelagic N:P ratio was near 40. As a result, the average summer surface chlorophyll (Chl) has exceeded 20 µg Chl/L only twice since 1986 [31].

In 2015–2018, samples from Butterfield Lake were tested for microcystins, cylindrospermopsins, and anatoxin-a through the CSLAP program [21]. One pelagic water sample tested positive for anatoxin-a in 2017, but this report was associated with a dense bloom of the chlorophyte *Spirogyra* leaving the source of this toxin uncertain. In 2016–2018, large plumes of *Mougeotia* were observed in the water column, likely associated with infusions of phosphorus from the hypolimnetic waters into surface waters during spring and fall turnover. Despite few reports of pelagic cyanobacteria toxins, there is a history of health issues associated with Butterfield Lake. In 2015, a CSLAP volunteer reported rashes on their arms after working on the lake [32]. In 2018, a dog became lethargic from an unknown element after swimming in a bay on the southeastern shore and eventually expired [33]. In 2017 and 2018, a resident living just south of the current Channel sample site reported health problems leading to hospitalization [33]. The causative factor of these health events, including whether cyanobacteria toxins were involved, remains unclear.

We had previously observed *M. wollei* in Butterfield Lake; however, the spatial extent of its distribution and its production of toxins was unknown. As pelagic cyanobacterial toxins did not appear to be associated with the reported health concerns, we investigated whether benthic cyanobacteria could be a contributing factor. Here, we describe the spatial, temporal, and between-site variation of *M. wollei* and its cyanotoxin production in Butterfield Lake, as well as a preliminary investigation into the environmental drivers associated with PSTs occurrence in *M. wollei*.

## 2. Results

### 2.1. Site Selection

Benthic rake collections made at 15 locations in shallow waters along the western and southern portions of the lake identified two sites with large *M. wollei* mats (Figure 1). No *M. wollei* was found at the other 13 sites. One site (Channel) was located at a man-made channel inside of a wetland that extended approximately 300 m long and was 5 m wide. This channel was used to connect the lake to several homes and a private boat launch and marina. A second site (Dock) was located at the southern end of the lake by the public boat launch and fishing dock. *M. wollei* at the Dock site was patchier and growing amongst more aquatic vegetation when compared to the Channel site. *M. wollei* at the Channel site was found growing on or in the muddy substrate, whereas *M. wollei* at the Dock site was found on the muddy substrate, on aquatic vegetation, and in detached clumps floating on or near the shoreline.

### 2.2. Comparison of Analytical Methods

Six samples, three from each site, were analyzed for PSTs by LC-MS/MS, ELISA, and HPLC with fluorescence detection (HPLC-FL). Higher concentrations of PSTs were found in Dock site samples relative to the Channel site samples using all three methods; however, the absolute concentrations as measured by the three analytical methods were markedly different (Table 1). The relative abundance of PST variants, as determined by HPLC-FL, was as follows: GTX-3 (0–12%), GTX-5 (25–50%), dcGTX-3 (<1%), dcGTX-2 (<1%), STX (0–3%), dcSTX (0–3%), LTX-2/3 (30–80%) and LTX-5 (3–30%). The presence of these PSTs was confirmed by LC-MS/MS [34]. The LTXs, along with GTX-3 and GTX-5, accounted for most of the observed PSTs. The other PSTs, including STX, fluctuated, but represented a small portion of the total PST concentrations. The toxin profiles were similar between the sites. HPLC-FL measured considerably higher toxin concentrations than ELISA or LC-MS/MS and thus HPLC-FL was used as the primary analytical method for these studies.

### 2.3. Species Identification

**Figure 2:**
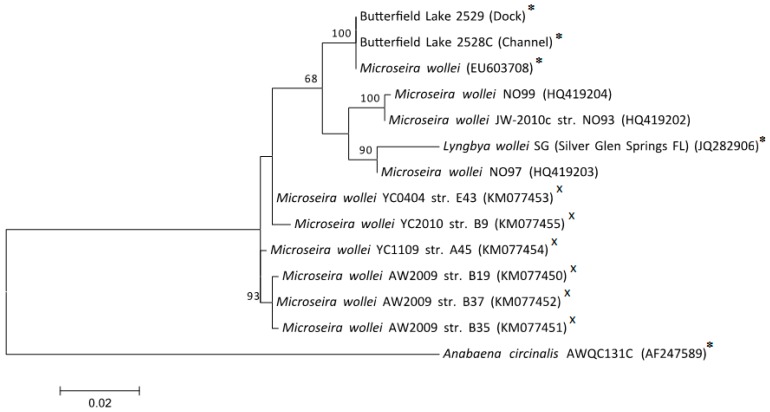
Maximum likelihood tree of 16S rRNA gene sequences from strains of *Microseira wollei*. Support values of 100 bootstrap iterations are shown at the nodes. Known paralytic shellfish poisoning toxin (PST) producers (✸) and cylindrospermopsin producers (**×**) are labeled; species without labels were not tested for toxins. *M. wollei EU603708* was the PST producer collected from Guntersville Reservoir, Alabama [5].

To determine if differences in PST occurrence at the two sites were associated with a difference species composition, 16S rRNA gene sequence analysis was conducted on *M. wollei* from the Dock and Channel sites. Samples from both sites yielded partial 16S rRNA gene sequences (682 and 678 at the Channel and Dock, respectively) that were identical over the 678 bp alignment. These sequences showed greater than 99% identity to 16S rRNA gene of *M. wollei* originating from the Guntersville Reservoir, a known PST producer [5]. In a maximum likelihood tree comprising 16S sequences from PST-producing strains of *M. wollei*, the Butterfield Lake sequences formed a highly supported cluster with the strain from Guntersville Reservoir (Figure 2).

### 2.4. Spatial Variation within the Channel

**Figure 3:**
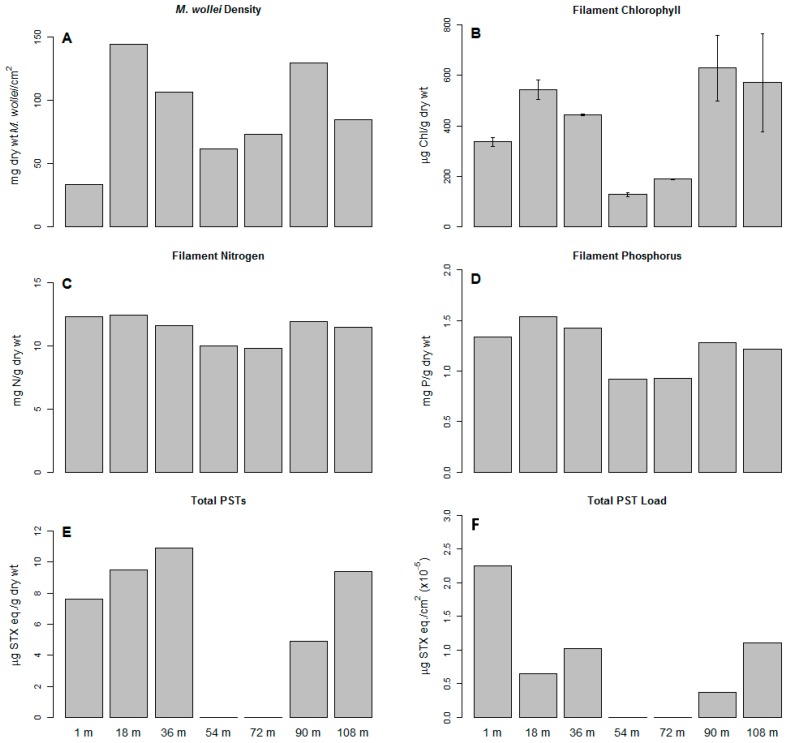
Spatial variation in different parameters related to *Microseira wollei* collected at 18 m intervals along a 108 m transect at the Channel site: (**A**) *M. wollei* density in mg dry wt., (**B**) filament chlorophyll content in μg Chl per g dry wt., (**C**) total nitrogen content of the filaments in mg N per g dry wt., (**D**) total phosphorus in the filament in mg P per gram dry wt., (**E**) total paralytic shellfish poisoning toxins (PSTs) in saxitoxin (STX) equivalent per g dry weight as determined by HPLC-FL, and (**F**) μg STX equivalents per square centimeter coverage of the benthos. Error bars for filament chlorophyll represent one standard deviation from duplicate measurements. Error bars for measurements of filament nitrogen, filament phosphorus, and total PSTs were <5% and are not shown.

Transects along the length of the Channel site demonstrated that *M. wollei* coated the bottom in a thick mat (Figure 3). *M. wollei* coverage per square centimeter varied along the Channel (Figure 3A) with an average of 90.8 ± 38.9 mg dry weight/cm^2^. Three replicates collected in close proximity varied by less than 10% in their dry weight. The *M. wollei* at transect locations 54 m and 72 m were associated with lower filament chlorophyll per g dry wt. (Figure 3B; 159 ± 43.0 µg Chl/g dry wt.) and contained no detectable PSTs (Figure 3C). These two sites had significantly less filament chlorophyll than the other five sites (average 505 ± 115 µg Chl/g dry wt.). There was little difference in the filament nitrogen (11.4 ± 1.1 mg N/g dry wt.) and filament phosphorus (1.23 ± 0.24 mg P/g dry wt.) of the *M. wollei* across all seven transect locations (Figure 3C,D). Five of the seven samples tested positive for PSTs (average 8.45 ± 2.30 µg STX eq./g dry wt.). PSTs/cm^2^ and Chl/cm^2^ were negatively correlated to biomass/cm^2^ (R^2^ = 0.9; *p* < 0.02) if the sites without detectable PSTs were excluded from the regression. The relationship between total PSTs per area or filament chlorophyll per area to biomass/cm^2^ was less clear when the 54 m and 72 m samples were included in the regression (R^2^: 0.14–0.16). None of the samples tested positive for microcystins, anatoxins or cylindrospermopsins.

### 2.5. Temporal Variation Between Sites

A comparison of the basic water quality parameters for the Dock and Channel sites are shown in Table 2. Both nutrient and physical parameters at the Channel and Dock were markedly different, with the Channel being 1.6 °C colder, a higher conductance, and a lower pH than at the Dock, as shown in Table 2. The calculated 1% light level for both sites reached the sediment surface of 1.5 m and 1.5–1.75 m for the Channel and Dock, respectively. Total phosphorus and total dissolved nitrogen in the Channel were elevated over the Dock by about 50%, and total nitrogen was elevated in the Channel over the Dock by about 30%. Physical parameters and pelagic nutrient concentrations at the Dock were representative of the conditions of the southern basin of the lake and CSLAP site (Table S1, Supplementary Materials).

**Figure 4:**
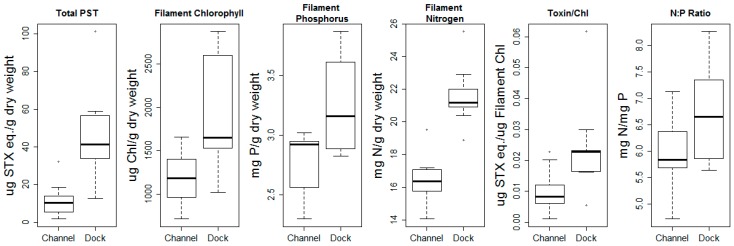
Box-and-whisker plots for six parameters measured in *Microseira wollei* filaments collected biweekly at both sites in 2017. Paired two-tailed *t*-tests were significantly different for all parameters. The *p*-values are adjusted using Holm’s correction with 8 degrees of freedom; tested parameter, difference in means, Holm’s adjusted *p*-value: (Total PST: 33.19, and 0.012); (filament chlorophyll: 778.78, and 0.026); (filament phosphorus: 0.50, and 0.005); (filament nitrogen: 5.25, and 0.0007); (toxin/chlorophyll: 0.014, and 0.026); (N:P ratio: 0.73, and 0.026).

*M. wollei* measurements included average filament chlorophyll, filament nitrogen, filament phosphorus, N:P ratio, total PSTs per g dry wt., and PSTs per chlorophyll. All were higher at the Dock site than at the Channel site for most of 2017 (Figure 4). Trace levels of anatoxin-a (0.008–0.07 µg anatoxin-a/g dry wt.) were occasionally detected in benthic mats at both sites, but no toxins were detected in water samples from either site. Anatoxin-a and PSTs were detected in the water column using Solid Phase Adsorption Toxin Tracking (SPATT) bags at both sites (21–513 µg STX eq./g resin and 0.04–1.2 µg anatoxin-a/kg resin), but again at trace levels. *M. wollei* in the Channel and Dock sites had weight-based N:P ratios of 5.92 ± 0.82 and 6.65 ± 0.92, respectively (Figure 4). The N:P ratios at both sites were lower than the weight-based Redfield ratio of 7.2:1 [35]. There were no differences in total carbon per g dry wt. *M. wollei* between the Channel and Dock sites (38.7 ± 1.95% and 38.7 ± 0.86%, respectively) [36,37], nor were there consistent differences in the summed concentrations of Al, Fe, Mg, and Ca in the *M. wollei* filaments, indicating variation in the degree of sediment contamination of the samples collected from the two sites was unlikely.

### 2.6. Environmental Factors as a Predictor of Total PSTs and Chlorophyll

**Figure 5:**
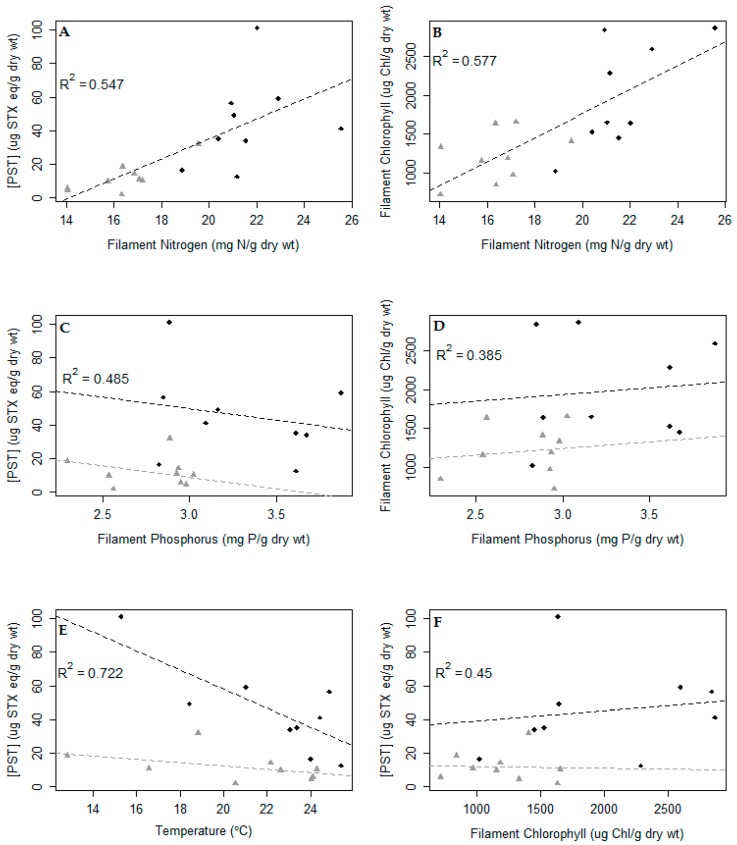
The relationships between filament nitrogen and total paralytic shellfish poisoning toxins (PSTs) (**A**) and filament chlorophyll (**B**), between filament phosphorus and total PSTs (**C**) and filament chlorophyll (**D**), between total PST and temperature (**E**) and between total PST and filament chlorophyll (**F**) using the best analysis of covariance (ANCOVA) model at the Dock (◆) and Channel (▲) sites. The maximal ANCOVA model of continuous predictor, site, and the interaction of site, and predictor were reduced to be minimally explanatory. Each point represents a composite sample collected every two weeks over the summer and fall.

Analysis of covariance (ANCOVA) regression models with either filament phosphorus, filament nitrogen or temperature as independent variables had complex relationships with site, filament chlorophyll and total PSTs (Figure 5). Filament nitrogen affected total PSTs and filament chlorophyll similarly at both sites (Figure 5A,B). Filament phosphorus (Figure 5C,D) had little or no impact on filament chlorophyll concentrations, but *M. wollei* at the Dock site had approximately 30% more filament chlorophyll per g dry wt. at the same concentration of filament phosphorus. There was a negative relationship between PSTs and filament phosphorus, and for the same concentrations of filament phosphorus, there were approximately 2.5 times more PSTs in *M. wollei* collected at the Dock site than at the Channel site. Temperature (Figure 5E) had a strong negative relationship with PSTs, and there was no relationship between temperature and filament chlorophyll (data not shown). The relationships between temperature and total PSTs were different for the two sites, with decreases in temperature at the Dock leading to a 5 fold greater increase in toxin than at the Channel site. Filament chlorophyll did not relate to total PSTs (Figure 5F), with the Channel having a weakly negative slope and the Dock having a weakly positive slope; neither of the slopes was significantly different from 0.

**Figure 6:**
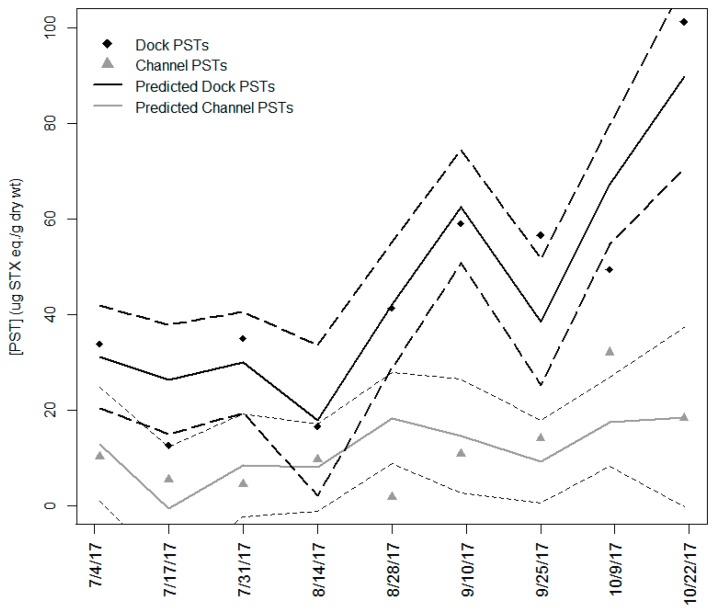
Total paralytic shellfish poisoning toxins (PSTs) and predicted PSTs from the final model of site, temperature, and filament chlorophyll (R^2^ = 0.858, *F*_4,13_ = 19.56). Dotted black and grey lines are the 95% confidence intervals for predicted total PST concentrations at each site. The full model of 8 terms was reduced to minimally adequate model using Akaike information criterion (AIC) and predicted residual error sum of squares (PRESS). Total PSTs on 17/07/2017, 25/09/2017, and 09/10/2017 fell outside the 95% CI for the model.

The minimally adequate model needed to predict total PST concentrations consisted of three parameters listed in their order of importance: site, temperature, and filament chlorophyll (Figure 6). The model contained these three terms with an interaction between temperature and site. By itself, filament nitrogen was a better predictor of PSTs when compared to filament chlorophyll, but these two prediction terms were covariate and the inclusion of filament chlorophyll over filament nitrogen produced a marginally better model (ΔAIC, 2.32; ΔPRESS, 405.98; ΔR^2^, 0.02).

## 3. Discussion

The choice of analytical method for PST analysis is non-trivial. Four analytical methods for PST have replaced the original mouse bioassay (AOAC 959.08): HPLC with fluorescence detection after pre-column chemical oxidation (AOAC 2005.06) [38] or post-column chemical oxidation (AOAC 2011.02) [39], LC-MS/MS (interlaboratory certification currently in process) [40,41,42], or an enzyme-linked immunosorbent assay (ELISA) [43]. All four methods have advantages and disadvantages as a primary analytical tool. The two oxidation methods convert the tricyclic PST ring system into a fluorescent derivative. Functional groups change the conversion efficiency, but most of the ~60 known PST analogs form a fluorescent product. LC-MS/MS is slowly being adopted for regulatory use with shellfish, but is limited by the availability of certified standards. Currently, standards are commercially available for 17 PSTs; sixteen of these standards correspond to marine PSTs, which may or may not be the predominant variants in freshwater systems. Only one freshwater PST, LTX-1, is commercially available as a certified standard. ELISA is a useful screening tool but is not recommended for quantification [44], as it provides no structural information about the PST variants in a sample, and most ELISA methods for PSTs were developed to monitor for shellfish toxins such as STX. The cross-reactivity to PST analogs present in cyanobacteria, including the LTXs, is highly variable if known at all.

Six *M. wollei* samples from Butterfield Lake were measured by post-column HPLC-FL, LC-MS/MS, and ELISA. As expected, HPLC-FL, with its ability to respond to a broader range of PSTs, reported a higher concentration of total PSTs than the ELISA assay. Concentrations of PSTs measured by LC-MS/MS analysis were also lower than total PSTs as measured by HPLC-FL. This was expected, as additional toxins such as LTX-2 through LTX-6 were present in the HPLC-FL analysis that were not included in the LC-MS/MS analysis. Despite these quantitative differences, the methods were internally consistent. Higher levels of PSTs were observed at the Dock site relative to the Channel site with all three techniques.

Because of the limited availability of freshwater PST standards and the abundance of the LTX analogs in our samples, we chose HPLC-FL with post-column oxidation as our primary analytical tool. One complication is that different analogs have different conversion efficiencies in forming fluorescent product(s). We adopted a single response factor based on STX, fully recognizing that some variants (GTX-2 and GTX-3) had a higher response factor, whereas other variants (GTX-1, GTX-4, and LTX-1) had a lower response factor relative to STX. In this approach, the HPLC-FL method was operated as an assay (as opposed to an analysis), mimicking the ELISA which quantified total PSTs in µg STX eq. using a single congener (STX) for the standard curve. This allowed us to estimate PST concentrations where standards were not available.

Butterfield Lake represents the second documented case of PST production by *M. wollei* in Northeastern USA and the first documented case of benthic production of PSTs within New York [21]. The concentrations of PSTs reported here were comparable to the total amounts of PSTs found by Hudon et al. in the St. Lawrence River [45]. *M. wollei* dominated in mats in Butterfield Lake and PSTs were present in most samples. *M. wollei* was the most likely source of the PSTs identified in Butterfield Lake based on the similarity of the 16S rRNA gene sequence of *M. wollei* from Butterfield Lake to the PST-producing *M. wollei* strain from Alabama, the similarity of the PST congener profiles between the Alabama *M. wollei* and the profiles observed in the two Butterfield Lake sites, and the dominance of *M. wollei* in the benthic mat from both sites. Genetic work to confirm the presence of the *stx* biosynthetic operon in the *M. wollei* from Butterfield Lake is currently in progress.

We tested for several other types of cyanobacteria toxins in Butterfield Lake. Only trace levels of anatoxin-a were detected in addition to the PSTs. There were no detectable levels microcystins, homo-anatoxin, cylindrospermopsins or deoxycylinderospermopsin in these samples. The source of anatoxin-a was uncertain. While *Anabaena* and *Phormidium* species were associated with anatoxin-a production in wadeable streams in California and New Zealand [46,47,48], these genera were not observed during microscopic examination of the cyanobacterial mats from Butterfield Lake. These and/or other anatoxin-a producing benthic cyanobacteria may have been present in the mat in small quantities and could account for the low levels of anatoxin-a. Regardless of the source, the low measured concentrations of anatoxin-a suggest there was little or no risk to lake residents and other users of the lake by toxins other than PSTs. Total PSTs concentrations ranged over the season but they were always the major cyanobacterial toxin found in Butterfield Lake.

We considered both intra- and extracellular toxin concentrations when evaluating the risk to humans from exposure to these toxins. Routes of exposure to PSTs in Butterfield Lake are comparable to their pelagic counterparts [24,49], where recreational exposure through bathing and/or drinking water dominate the discussion of pelagic blooms [50]. In Butterfield Lake, recreational or other direct contact with *M. wollei* was more likely to occur at the Dock site than at the Channel site due to its proximity to people. However, even at the Dock site, most *M. wollei* was located far enough from the shoreline to limit potential human contact. Unlike pelagic cyanobacteria blooms, swimmers were unlikely to encounter the toxic benthic mats unless they detached from the bottom substrate. Additionally, the highest toxin concentrations occurred later in the season, coinciding with decreasing water temperatures. Lower temperatures would correspond with less recreation at the lake, further decreasing the risk of direct exposure to *M. wollei* containing high levels of toxin.

A second route of exposure is through consumption of drinking water contaminated by PSTs. Many lake residents pump water directly from the lake into their homes. The residents use different approaches to treat their water, with filtration being the most common but some using UV-disinfection or chlorination [33,51]. PSTs are quite polar and residential charcoal filtration units, generally designed for non-polar taste and odor compounds, are not very effective at removing PSTs from the water. Dissolved toxins were never detected in grab samples from either the Channel or the Dock sites; anatoxin-a and PSTs were only detected in SPATT bags that were deployed in the water column for two weeks. The concentrations of anatoxin-a and PSTs in the water were always lower than the recommended regulatory limit for these toxins, suggesting risk from direct exposure in drinking water was low [24,52,53].

The presence of benthic cyanobacteria mats in Butterfield Lake was very consistent at the two sites but offers a different challenge for monitoring when compared to a traditional pelagic bloom. The New York CSLAP program is well suited to monitoring pelagic algal blooms and their corresponding toxins; using a combination of temporal sampling over the growing season and spot sampling of visible blooms to track the temporal and spatial variation of the bloom. Due to its original purpose as a monitoring program for tracking long-term changes in water quality, CSLAP samples are usually collected from a limited number of open water sites in the center of the lake. A similar approach was used in the EPA National Lakes Assessment to evaluate cyanobacterial toxins [54]. These generic procedures are not well suited to monitoring for cyanotoxins from benthic mats where the choice of sampling site becomes increasingly important. If collections were made in Butterfield Lake at only the Channel or Dock sites, or any of the 13 other sites from around the lake, it would have resulted in an incomplete assessment of toxin content in Butterfield Lake. Extrapolating toxin content to an entire waterbody from a limited number of samples would therefore potentially misrepresent toxin concentrations and their exposure risk.

The choice of site was the most important parameter in our multivariate models when evaluating what factors influenced the presence of PSTs. These parameters were different from those derived for pelagic cyanobacterial blooms. While Butterfield Lake was phosphorus-limited in the surface water with a weight-based N:P ratio around 40, *M. wollei* filaments were generally nitrogen-limited, with N:P ratios below 7.2 for the majority of samples. Only three samples, all at the Dock, had N:P ratios above the Redfield ratio. Changes in filament nitrogen content were closely linked to changes in total PSTs and filament chlorophyll, while filament phosphorus content had no relationship with filament chlorophyll at either site. Benthic cyanobacteria grow in close proximity to the sediments and phosphorus content in Butterfield Lake sediments can be quite high (420 mg P/kg sediment [33]). Thus *M. wollei* could acquire the phosphorus needed for growth directly from the sediments, even when surface water phosphorus concentrations were low [55]. Unlike benthic cyanobacteria found in the Eel River [56], *M. wollei* in Butterfield Lake was never phosphorus-limited. In contrast, sediment N was low (<110 mg N/kg sediment [33]) and filament nitrogen was closely related to both total PSTs and filament chlorophyll. These results are very different from those results obtained in culture using *M. wollei* isolated from Alabama where both high nitrate and high phosphate resulted in a marked decrease in PSP toxicity [57].

Despite this lack of phosphorus limitation, there was still a weak negative relationship between total PSTs and filament phosphorus. Boyer et al. [58] observed a similar trend in dinoflagellates grown under phosphorus-limited conditions, where decreasing phosphorus increased the toxicity per cell. A similar trend was observed here with *M. wollei*. Boyer et al. attributed this increase to a decreased growth rate that in turn led to an increase in toxin content per cell. A similar relationship may occur with *M. wollei* in Butterfield Lake.

The relationships between PSTs, environmental parameters, and site were diverse. Still the choice of site remained the most important variable in explaining the PST concentrations in Butterfield Lake. The similarity in 16S rRNA segments between the Dock site and Channel site indicate that changes in toxin production were not due to changes in species, but that environmental and/or biological differences between the sites may be important. Although there were important relationships between nutrients and total PSTs, models to predict total PST content did not include the filament nutrient concentrations as predictors, only environmental variables in addition to filament chlorophyll. Filament chlorophyll, which may reflect filament nitrogen content, was a better predictor of total PSTs than the filament nitrogen content itself. Toxin concentration was primarily driven by site, temperature, and filament chlorophyll, with significant loss in explanatory power when each of these terms was removed. Calcium content, an important predictor of biomass and toxicity in culture [57], was not different between the two sites and could not explain the observed differences. Temperature effects were highly dependent on the site. As temperatures dropped later in the season, total PSTs, both measured and predicted by the model, diverged at the two sites; decreases in temperature had a larger impact on PST concentrations at Dock site relative to the Channel site. Filament PST content at the two sites were similar early in the season, but the total PST content increased much more at the Dock site than at the Channel site as the season extended into September and October. The cause of the differences between the sites are unknown, but might be linked to the close association of *M. wollei* with submerged aquatic vegetation at the Dock site, where aquatic vegetation was largely absent in the Channel.

The environmental drivers of *M. wollei* PSTs were interesting in the context of freshwater pelagic blooms, where pelagic bloom size is closely related to phosphorus, while microcystins and anatoxin-a content is more closely related to nitrogen availability [59,60,61,62,63] and genetic factors [64]. The drivers of benthic toxin production may not be the same as those for pelagic cyanobacteria, with genetic factors, physical parameters, and nutrient parameters all playing important roles [47,65]. This neglects the important role sediments play as a source of nutrients to the cyanobacteria. Hudon et al. [45] collected *Lyngbya* (potentially *Microseira)* samples from two large fluvial lakes in the St. Lawrence River and examined the correlation of several environmental parameters to LTX-1. They identified depth, dissolved organic carbon, and the 1% light level as important contributors for toxin occurrence, while water flow was important in mat proliferation. Water flow did not change in Butterfield Lake, while the 1% light level was at or below the sediment water interface at both sites. Further studies would be needed to better define the role of light in this lacustrine system. Hudon et al. reported no significant differences in LTX-1 concentrations between their two fluvial lakes. This was very different from what we observed in Butterfield Lake where LTX-1 was absent and there was a large variation in the other lyngbyatoxins. This emphasizes the importance of a representative and balanced sample design that employs a broad-spectrum analytical technique and multiple sites when trying to assess the overall risk from benthic PSTs.

## 4. Methods

### 4.1. Sample Collection and Processing

Ten locations along the southwestern shoreline and five locations on the eastern shoreline of the lake were investigated for the presence of *M. wollei*. The collections sites were 50–300 m apart on the western shore, and 300–1000 m apart on the eastern shore. At each of these 15 locations, 1–5 rakes were tossed in different directions from the boat, dragged across the bottom and handsorted back in the boat to find *M. wollei*. For temporal analysis, the rake was tossed three times into different portions of the *M. wollei* mat and a ~10 g wet weight portion of cyanobacteria from each toss was combined to form one sample. Samples were collected every two weeks between July and October. Plant material and detritus were handpicked out from the samples of *M. wollei*, and the sample was washed in the field to remove sediments. For spatial coverage, a petite ponar (Wildco, Yulee, FL, USA) was dropped vertically into the *M. wollei* mat and the resulting mud mixture/biomass mixture was placed into a bucket. Variation due to sampling technique was evaluated by collecting three replicates in close proximity. Cyanobacteria mat material was picked out by hand, washed using lake water and returned to the lab. In the lab, the samples were cleaned again to further remove sediment. Both rake and ponar samples were placed on ice until their return to the lab. Once in the lab, the samples were weighed, immediately frozen at −80 °C, and freeze-dried. The freeze-dried samples were homogenized in a mortar and pestle using liquid nitrogen, and the powder stored at −20 °C until further analysis. Measurements of water column chlorophyll, phycocyanin, temperature, pH, and conductivity were collected with a Hydrolab DS 5X Sonde, (HACH Environmental, Loveland, CO, USA) at each collection site.

### 4.2. Nutrient Analyses

Filament total nitrogen and phosphorus were measured in duplicate on ~30 mg of portions of lyophilized *M. wollei* powder. Material was fragmented by sonification (3 × 20 s at 32 watts), and a ~20 mL aliquot sonicated further (12 × 20 s at 32 watts) to lyse the filament prior to analysis. Lake water was collected and maintained in the field at 4 °C for analysis of total phosphorus and total nitrogen, or sterile filtered (0.22 µm) in the field and maintained at −20 °C for later analysis of total dissolved nitrogen. Total phosphorus (EPA method 365.1) and total nitrogen (EPA method 353.2) were measured using these samples using a SEAL autoanalyzer model AA3 (SEAL Analytical, Mequon, WI, USA).

### 4.3. Other Analyses

Chlorophyll-a was determined by extraction of duplicate lyophilized powders using a modification of EPA method 445.0 and analyzed using a Turner Design TD-700 fluorimeter (Turner Design, San Jose, CA, USA). Total carbon was determined on ~2 mg of sample with a Thermo Scientific FlashEA 1112 elemental analyzer (Thermo Scientific, Waltham, MA, USA) interfaced with a thermal conductivity detector. Filament-associated metal content was determined by ICP-OES (Perkin Elmer Optima 3300DV, Waltham, MA, USA). Approximately 50 mg of *M. wollei* powder was heated to 75 °C overnight in 10 mL concentrated nitric acid. Samples were then centrifuged at 10,000× *g* for 5 min, diluted to a final concentration of 7.5% nitric acid, and infused into the ICP-OES (Perkin Elmer Optima 3300DV) for measurement of the common soil elements: iron, aluminum, calcium, magnesium, and sulfur.

### 4.4. DNA Extraction and Analysis

Genomic DNA was extracted from freeze-dried environmental samples using a method modified from Kurmayer et al. [66]. Ten milligrams of freeze-dried cells were incubated in 750 µL of hydration/osmotic shock buffer containing 100 mM EDTA, 50 mM Tris-HCl (pH 8.0), and 25% (*w*/*v*) sucrose for 2 h on ice. Lysozyme (25 µL of 100 mg mL^−1^) was added and incubated at 37 °C for 20 min. Proteinase K (50 µL of 1 mg mL^−1^) and 50 µL of 10% SDS (*w*/*v*) were added and incubated at 50 °C for 2 h. DNA was collected with three extractions using phenol/chloroform/isoamyl alcohol (25:24:1, *v*/*v*/*v*) followed by two extractions of chloroform/isoamyl alcohol (24:1, *v*/*v*). DNA was precipitated with sodium acetate (0.3 M) and 100% ethanol, and then washed with 70% ethanol using standard methods [67]. DNA was quantified with a NanoDrop ND-1000 spectrophotometer (Thermo Scientific, Waltham, MA, USA) and stored at −20 °C until used as a template in PCR. Partial sequence of the 16S rRNA gene was amplified using the cyanobacteria-specific primer set 27F and 809R [68]. PCR reaction mixtures were formulated with EconoTaq Plus Green 2× Master Mix (Lucigen, Middleton, WI, USA) using ~30–50 ng of genomic DNA as a template. Thermal cycling conditions were 94 °C for 2 min, followed by 37 cycles of 94 °C for 30 s, 64 °C for 30 s, and 72 °C for 90 s, followed by a final extension cycle of 72 °C for 5 min. The PCR product size was verified in agarose gels. PCR products were cleaned using the QIAquick PCR purification kit (Qiagen, Hilden, North Rhine-Westphalia, Germany). PCR products were sequenced on the Applied Biosystems 3730 Genetic Analyzer (Thermo Scientific, Waltham, MA, USA) at the Genomics Core Facility at the University of Tennessee, Knoxville. Phylogenetic analysis and tree construction were conducted in MEGA7 [69]. Sequences were aligned using MUSCLE and default settings. Alignment gaps were deleted. Trees were constructed using the maximum likelihood method based on the general time reversible model and a discrete Gamma distribution with five categories. Support for tree topology was assessed with 100 bootstrap iterations.

### 4.5. Toxin Extraction and Analysis

Microcystins, cylindrospermopsins, anatoxins, and PSTs were extracted 50% methanol containing 1% acetic acid (*v*/*v*). Ten milliliters was added to ~700 mg of cyanobacterial powder prior to probe sonication (3 × 20 s at 32 watts) on ice. The slurry was centrifuged at 15,000× *g* for 10 min, passed through a 0.45 µm nylon syringe filter, and was the same sample used for HPLC-FL, ELISA, LC-MS, and LC-MS/MS analyses. SPATT bags were constructed as described in Lane et al. [70], using 5 g of DIAION HP20 resin. SPATT bags were deployed 1 m under the surface of the water at the Channel and Dock sites for two weeks. Toxins were extracted from the resin using 100% MeOH. The solvent was removed in vacuum and the sample reconstituted into 2 mL of distilled water. Microcystins were analyzed by LC-MS as described in Tang et al. [62]. Anatoxin-a and homo-anatoxin-a were analyzed by LC-MS/MS using a modified version of EPA method 545 that included one quantification and two confirmation ions for each toxin. Cylindrospermopsin, epi-cylindrospermopsin and deoxycylinderospermopsin were determined by LC-MS/MS in the same run again using one quantification ion and two confirmation ions [45]. PSTs were analyzed using AOAC 2011.02 post-column chemical oxidation modified for water samples and algal powders [71]. Separation used a Waters Alliance 2695 solvent delivery system (Waters, Milford, MA, USA), and a Chromenta KB 3µ 150 × 4.6 mm column with an ACE (ACE Ltd., Aberdeen, Scotland, UK) 3µ guard cartridge assembly at 0.8 mL/min. The solvent system was: A, 2 mM heptanesulfonate (Regis Technologies Inc., Morton Grove, IL, USA) in 10 mM ammonium phosphate adjusted to pH 7.1; B, 500 mL 2 mM heptane sulfonate in 30 mM ammonium phosphate adjusted to pH 7.1 + 150 mL of acetonitrile [72]. The separation gradient was: 0% B for 0–3 min, 40% B for 3–5 min, 100% B for 5–13 min, and 100% B for 20 min, followed by equilibration of the column back to 0% B for 10.5 min. Oxidation of the PST ring used 9 mM periodic acid (Alfa Aesar, Ward Hill, MA, USA) in 50 mM potassium phosphate at pH 9 in a reaction coil temperature maintained at 65 °C. The acid modifier was 0.5 M acetic acid with a flow of 0.45 mL/min. PSTs were differentiated from interfering fluorescent compounds by re-injection of the sample with water in place of the oxidant. Individual method LODs were determined for each toxin from their average daily response factors. Microcystin LODs averaged 0.20 µg/g and 0.50 µg/L for *M. wollei* samples and whole water samples, respectively. Method LODs for anatoxin-a and homo-anatoxin were 0.005 µg/g and 0.02 µg/L for *M. wollei* and whole water samples, respectively. Cylindrospermopsin and deoxycylinderospermopsin LODs were 0.05 µg/g and 0.1 µg/L, respectively. PST LODs were 0.15 µg/g and 0.10 µg/L, respectively. Primary PST standards were purchased from the NRC Canada (Institute for Marine Biosciences, Halifax, Canada) and United States Food and Drug Administration (FDA) (Silver Spring, MD, USA). FDA STX was diluted 1:50 to a concentration of 4 µM prior to use. STX standard response converted into an equivalent *M. wollei* concentration was linear between 1.3 and 184.5 µg/g using five points. NRC standards of STX (66.3 µM), dcSTX (2.6 µM), GTX-1 (2.3 µM), GTX-2 (4.1 µM), GTX-3 (1.7 µM), GTX-4 (0.7 µM), GTX-5 (2.3 µM), dcGTX-2 (4.0 µM), dcGTX-3 (1.2 µM), LTX-1 (17 µM), and C1+C2 (5.9 µM) were used to calculate relative response factors. Uncalibrated neosaxitoxin was used as a retention time standard. Response factors for other variants of PSTs relative to STX ranged from 0.1 to 4.6 with an average of 1.44. All PSTs, both known and unknown, were quantified using an STX standard curve with single injections of STX standards to verify the stability of the response factor. For comparison of analytical methods, three samples were chosen from the Dock site and three samples were chosen from the Channel site, with three samples from the beginning, middle, and end of the sampling period each. PSTs were analyzed by LC-MS/MS per the method described in Armstrong et al. [73] using a Waters Xevo TQD mass spectrometer (Waters, Milford, MA, USA) in the laboratory of Professor Juliette Smith at the Virginia Institute of Marine Science, and by Pearse McCarron at the National Research Council of Canada. STX was determined using Abraxis ELISA (part number 52255B, Abraxis LLC, Warminster, PA, USA) according to the manufacturer’s instructions. Samples for ELISA were diluted to achieve a maximum methanol concentration of <5% prior to analysis.

### 4.6. Statistical Analysis and Model Selection

Statistical analyses were performed in R version 3.5.1 (R Core Team, Vienna, Austria) with base package tools. Multiple comparisons for paired *t*-test *p*-values were corrected with Holm’s stepdown procedure (*n* = 6 parameters). Durbin–Watson tests assessed autocorrelation from temporal pseudoreplication in parameters used in the ANCOVA and multivariate models, with significant autocorrelation found only in total PSTs collected from the Channel (*p* < 0.05). Small deviations from normality were observed in the residuals for some tests, but were not deemed sufficient enough to warrant transformation. Transformation did not change the significance or interpretation of the tests or results, with the loss of information in the magnitude of differences deemed excessively detrimental. ANCOVA maximal models started with predictor, site, and predictor site interaction, and were reduced to a minimum explanatory model using *F* tests (*p* < 0.05). The explanation of the variation in PSTs from both sites was performed by a multivariate linear regression containing biological and environmental variables collected from the *M. wollei* and the surrounding water. The full model started with the following predictors: filament chlorophyll, filament nitrogen and phosphorus, temperature, site, temperature/site interaction, and CSLAP water column total phosphorus and total nitrogen, and was simplified to be minimally adequate using manual and automated forwards and backwards stepwise addition or removal of parameters. Model simplification was done by a combination of *F* tests and AIC (*p* < 0.05, ΔAIC = 2). Coefficients for the final model were intercept (25.71), temperature (−1.53), site (149.92), chlorophyll (0.015), and interaction (−5.66). Final model statistics (R^2^ = 0.858, *F*_4,13_ = 19.56). Filament chlorophyll and filament nitrogen were covariate so only one term was kept in the reduced model. Non-nested models containing either filament chlorophyll or filament nitrogen were compared using Akaike information criterion (AIC) and predicted residual error sum of squares (PRESS) [74]. Relative importance of the terms in the final model was evaluated by comparison of each nested model to the final model by AIC and PRESS. Site, temperature, and filament chlorophyll explained a majority of the variation in total PSTs over 2017.

## Figures and Tables

**Table 1:** Paralytic shellfish poisoning toxin (PST) concentrations measured in six samples by three different analytical methods.

Sample Date	Dock			Channel		
	HPLC-FL ^1^	ELISA ^1^	LC-MS/MS ^2^	HPLC-FL ^1^	ELISA ^1^	LC-MS/MS ^2^
7/4/2017	33.77	6.94	22.33	10.23	5.75	3.63
9/10/2017	58.98	4.18	20.12	10.81	3.27	4.85
10/22/2017	101.25	36.24	37.95	16.00	2.58	9.56

^1^ Total PSTs calculated in µg saxitoxin (STX) eq./g dry wt. ^2^ LC-MS/MS PSTs were quantified using 12 common marine PST standards. Standards were not available for the lyngbyatoxins (LTXs) so the contributions of these toxins to the total PST pool as measured by LC-MS/MS were not included.

**Table 2:** Average water quality parameters for the Dock and Channel sites measured between 4 July and 22 October, 2017 (*n* = 9). Temperature, conductivity, and pH were measured every two weeks. Water column nutrients were measured three times over the 4 month period. Light attenuation and depth were measured once in the middle and once at the end of the July-to-October sampling period.

Parameter	Dock	Channel
Depth (m)	1.5–1.75	1.5
Average Temperature (°C)	22.22 ± 3.38	20.66 ± 3.94
Conductivity (μS)	197.92 ± 19.25	225.61 ± 17.51
pH	8.27 ± 0.69	7.14 ± 0.28
TP (pelagic) (mg P/L)	0.0112 ± 0.0035	0.0215 ± 0.0038
TDP (pelagic) (mg P/L)	Below detection *	Below detection *
TN (pelagic) (mg N/L)	0.320 ± 0.009	0.482 ± 0.077
TDN (pelagic) (mg N/L)	0.254 ± 0.009	0.419 ± 0.028
Light Attenuation Coefficient (k)	−1.87 ± 1.16	−2.12 ± 0.40
Calculated 1% light level (m)	1.51–6.48	1.83–2.68

* LOD < 0.009 mg P/L. TP, total phosphorus. TDP, total dissolved phosphorus. TN, total nitrogen. TDN, total dissolved nitrogen.

## Supplementary Materials

The following are available online at http://www.mdpi.com/2072-6651/11/1/44/s1, Table S1: Lake-wide nutrient measurements.
