# Supplementary material for: Spatial and Temporal Variation in Paralytic Shellfish Toxin Production by Benthic Microseira (Lyngbya) wollei in a Freshwater New York Lake
Source: Toxins (Basel). 2019 Jan 15;11(1):44. doi: 10.3390/toxins11010044 (PMC6356249; doi:10.3390/toxins11010044)
Supplement: Supplementary file 1 [file toxins-11-00044-s001.pdf]

Supplementary Materials: Spatial and Temporal Variation in Paralytic Shellfish Toxin Production by Benthic *Microseira (Lyngbya) wollei* in a Freshwater New York Lake

Zacharias J. Smith, Robbie M. Martin, Bofan Wei, Steven W. Wilhelm and Gregory L. Boyer

Table S1. Lake-wide nutrient measurements.

Sample Location	Total Phosphorus	Total Nitrogen	Total Dissolved Nitrogen
44°18′30.3″ N 75°47′14.4″ W	0.0079	0.314	0.269
44°18′42.9″ N 75°47′07.3″ W	0.0088	0.348	0.279
44°19′09.3″ N 75°47′13.3″ W (Channel)	0.0249	0.567	0.443
44°19′57.8″ N 75°45′57.7″ W	0.0143	0.404	0.293
44°20′19.9″ N 75°45′21.0″ W	0.0095	0.434	0.327
44°18′56.2″ N 75°46′33.4″ W	0.0043	0.323	0.289
44°18′06.5″ N 75°46′56.4″ W (CSLAP)	0.0112	0.298	0.238
44°17′58.8″ N 75°47′39.5″ W (Dock)	0.0080	0.309	0.243
